# Associations between Selected *ADRB1* and *CYP2D6* Gene Polymorphisms in Children with Ventricular and Supraventricular Arrhythmias

**DOI:** 10.3390/medicina59122057

**Published:** 2023-11-21

**Authors:** Ewa Moric-Janiszewska, Sławomir Smolik, Lesław Szydłowski, Małgorzata Kapral

**Affiliations:** 1 Department of Biochemistry, Faculty of Pharmaceutical Sciences in Sosnowiec, Medical University of Silesia in Katowice, Jedności 8B, 41-200 Sosnowiec, Poland; 2 Department of Pediatric Cardiology, Faculty of Medical Sciences in Katowice, Medical University of Silesia in Katowice, Medyków 16, 40-752 Katowice, Poland

**Keywords:** genetic polymorphism, β-blockers, pharmacogenetics

## Abstract

*Background and Objectives*: Tachycardia is a common cardiovascular disease. Drugs blocking β1-adrenergic receptors (ADRB1) are used in the therapy of arrhythmogenic heart diseases. Disease-related polymorphisms can be observed within the *ADRB1* gene. The two most important are Ser49Gly and Arg389Gly, and they influence the treatment efficacy. The family of the cytochrome P450 system consists of the isoenzyme CYP2D6 (Debrisoquine 4-hydroxylase), which is involved in phase I metabolism of almost 25% of clinically important drugs, including antiarrhythmic drugs. A study was conducted to detect the *ADRB1* and *CYP2D6* gene polymorphisms. *Materials and Methods*: The material for the test was whole blood from 30 patients with ventricular and supraventricular tachycardia and 20 controls. The samples were obtained from the Department of Pediatric Cardiology. The first to be made was the extraction of DNA using a GeneMATRIX Quick Blood DNA Purification Kit from EURx. The selected *ADRB1* and *CYP2D6* gene polymorphisms were detected by high-resolution melting polymerase chain reaction (HRM-PCR) analysis. *Results*: Based on the analysis of melt profile data for each PCR product, the identification of polymorphisms was carried out. Heterozygotes and homozygotes were found in the examined alleles. *Conclusions*: The frequency of the Arg389Gly polymorphism differs statistically significantly between the control group and patients with supraventricular and ventricular arrhythmias, as well as between these two groups of patients. Moreover, the Arg389Gly polymorphism was statistically more prevalent in the group of girls with SVT arrhythmia compared to girls with VT. A few carriers of homozygous and heterozygous systems of the S49G polymorphism were detected among patients with arrhythmias, as well as control group. The percentage of individuals carrying the *CYP2D6 4* allele as either homozygous or heterozygous was observed in the study and control groups. The high prevalence of the CYP2D6*4 allele carriers in both groups prompts the optimization of beta-1 blocker therapy.

## 1. Introduction

Cardiac arrhythmias represent often diagnosed ailments of the circulatory system, also in the group of children. The treatment includes drugs blocking β1-adrenergic receptors (ADRB1). However, there may be polymorphisms in receptor genes that may alter their function. Therefore, it is expected that patients using drugs (medicaments) that affect β1-adrenergic receptors may show different responses to the administered doses of the preparation. Numerous scientific studies have confirmed the relationship between the polymorphism of this receptor and the response to the therapy [1,2,3,4,5]. Some studies indicate that methylation of the β1 receptor is a potential epigenetic mechanism controlling response to therapy. Jiang Q. et al. [6] indicated that adrenergic beta 1 receptor (ADRB1) gene polymorphisms influence the level of response to metoprolol drug therapy, downregulating methylation of the Adrb1 promoter and improving its efficacy [6,7]. It should be noted that polymorphisms occur in the case of many other genes. Their detection makes it possible to learn about the behavior of a given receptor or enzyme in a given genotype. The discovery of all relationships between the type of polymorphism and the body’s response gives great opportunities for treatment development. Defining a patient’s genetic profile gives great hope for personalizing pharmacotherapy, which would bring considerable benefits [8,9].

Adrenergic receptors (ARs) belong to the amine receptor cluster of the rhodopsin-like family of G protein-coupled receptors (GPCRs). These receptors are present in almost all peripheral tissues and within the central and sympathetic nervous system. ARs constitute important drug targets; for instance, β1AR inverse agonists and antagonists are widely used to treat hypertension and heart disease [10].

Today, nine AR subtypes have been identified and classified into three major types based on pharmacological and molecular evidence, namely, α1, α2, and βARs, with three subtypes in each class [11,12]. β-adrenergic receptors are members of the G protein-coupled receptor (GPCR) superfamily whose signaling plays a critical role in regulating the function and processes of the cardiovascular system. Presently, three subtypes of β-ARs have been characterized (β1-AR, β2-AR, and β3-AR), with the fourth (β4-AR) remaining controversial [13,14,15]. The three subtypes have different affinities for different ligands, which allow variable activation of each subtype [15,16,17]. A healthy human heart has approximately a 4:1 ratio of β1-AR to β2-AR, with minimal expression of β3-AR [16,18].

**Figure 1:**
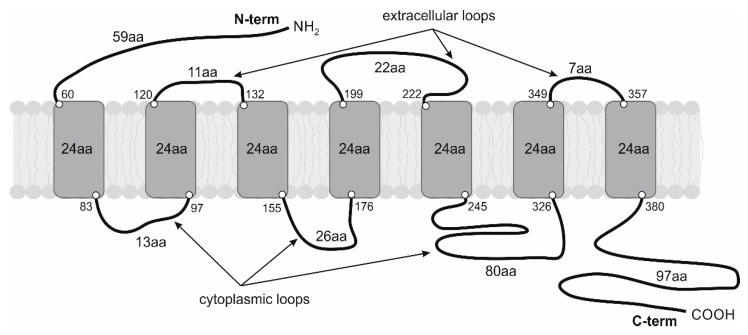
Structure of human β1 receptor.

It has recently been confirmed that β1-ARs are present in all cardiomyocytes [19]. On the other side, β1-AR is expressed at a low level in non-myocytes (endothelial cells) [19]. β-ARs, like other GPCRs, consist of a seven-transmembrane-spanning receptor and are coupled to an intracellular heterotrimeric G-protein complex [20]. See Figure 1.

β1-adrenergic receptor antagonists are a group of drugs with a wide pharmacological application used to treat cardiac arrhythmias, hypertension, and glaucoma. The patient response to the action of this group of drugs is conditioned by many genetic factors related to both the polymorphism of the drug target point and the enzymes involved in their metabolism. Among the many polymorphic sites identified within the β1-adrenergic receptor, the Arg389Gly and Ser49Gly polymorphisms have the highest frequency and clinical significance. The change of adenine to guanine at the 145th position results in the change of the amino acid serine to glycine (Ser49Gly), and the substitution of cytosine with guanine results in the change of the amino acid glycine to arginine (Gly389Arg). Familiarization with the genetic profile of patients makes it possible to predict the pharmacological response and select the drug dose [21,22].

The above polymorphisms influenced the pharmacological response to beta blockers used in the same doses, which, depending on the patient’s genotype, resulted in a weaker or stronger effect of the drug. Lower adenosine cyclase activity upon agonist simulation in heart samples from HF patients with the Arg389 allele than with the Gly389 allele was confirmed. Research has shown greater LVEF improvement in Arg389Arg than Gly carriers for metoprolol and carvedilol, there is no association for bisoprolol. However, the results have been somewhat inconclusive [1,2,5,21,23,24].

Cytochrome P450 2D6 (CYP2D6) is an enzyme that in humans is encoded by the *CYP2D6* gene. *CYP2D6* is primarily expressed in the liver. It is also highly expressed in areas of the central nervous system, including the substantia nigra. CYP2D6, a member of the cytochrome P450 mixed-function oxidase system, is one of the most important enzymes involved in the metabolism of xenobiotics, including antiarrhythmic drugs in the body. In particular, CYP2D6 is responsible for the metabolism and elimination of approximately 25% of clinically used drugs by adding or removing certain functional groups—specifically, hydroxylation, demethylation, and dealkylation [25,26,27,28].

**Figure 2:**
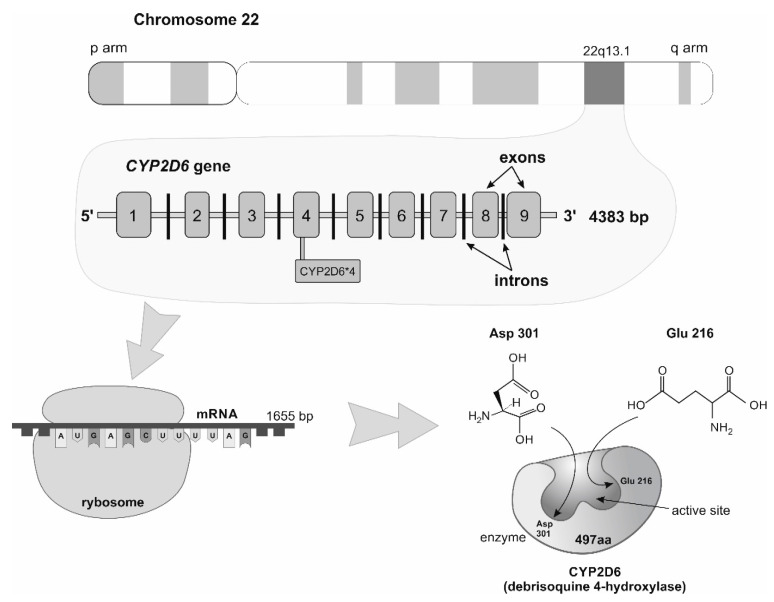
*CYP2D6* gene localization, structure, mRNA, and protein product (enzyme debrisoquine 4-hydroxylase).

The family of the cytochrome P450 system consists of the isoenzyme CYP2D6 (Debrisoquine 4-hydroxylase), the most representative in liver tissue (6% of all CYP450: CYP3A4, CYP2C9, and others) [29,30] is involved in phase I metabolism of almost 25% of clinically important drugs [31,32,33]. The autosomal *CYP2D6* gene is located on the long arm of chromosome 22 fragment q 13.1 (22q13.1); it consists of 4383 bp [34] grouped in nine exons and eight introns that produces an mRNA of 1655 bp [35,36], with a 1383 bp open reading frame encoding a 497 amino acid protein [29,37]. CYP2D6*4 (g.1846G > A, rs3892097) is characterized by a single-nucleotide polymorphism (transition from guanine to adenine) at position 1846 (first nucleotide) of exon 4, causing a truncated protein due to a defect in splicing [38,39,40,41]. See Figure 2.

The main aim of the study was to genotype two polymorphic sites Arg389Gly and Ser49Gly in the β1-adrenergic receptor gene and identify the polymorphism of the CYP2D6*4 gene in a group of 30 children with diagnosed cardiac arrhythmias and 20 non-arrhythmic controls to analyze the frequency of occurrence of the genotypes tested in the study, also taking into account gender and age.

## 2. Materials and Methods

### 2.1. Research Population

The research population consisted of 50 children diagnosed in the Department of Pediatric Cardiology of the hospital in Katowice. Thirty of them (14 boys, and 16 girls) were suffering from supraventricular (*n* = 11) or ventricular arrhythmias (*n* = 19) which were confirmed by interview, clinical examination and 24 h Holter ECG monitoring. The remaining 20 (arrhythmia-free) patients were in the control group (8 boys, and 12 girls). Arrhythmia was excluded by Holter ECG monitoring. Most of them were children admitted to the emergency department due to hypertension, chest pain and fainting, and these ailments did not have a cardiac etiology. Patients were aged 11 to 18 years (average age 15.5). All children underwent basic blood tests, ECG, and blood pressure measurements.

### 2.2. Serum Collection and Storage

Blood samples for HRM analysis were collected from the patients in the Department of Pediatric Cardiology. About 2.5 mL of blood for EDTA were collected. For this purpose, commercially available S-Monovette tubes (Sarstedt, Stare Babice, Poland) were used.

### 2.3. Genomic DNA Isolation

The GeneMATRIX Quick Blood DNA Purification Kit (v. 1.4 EURx, Gdańsk, Poland) with synthetic DNA binding membranes with the use of SiO2 silica oxide in spin columns was used. The concentration of genomic DNA was determined spectrophotometrically based on absorbance values at a wavelength of 260 nm using a UV-1800 spectrophotometer (Shimadzu, Duisburg, Germany). DNA purity was assessed by the ratio of absorbance at 260 and 280 nm (A260/A280) (ratios of 1.8–2.0 were acceptable). The qualitative evaluation of DNA was carried out on a 0.6% agarose gel stained with SimplySafe ™ dye, which emits green fluorescence under UV light and, due to binding to DNA, allows the assessment of integrity. Following isolation, samples were stored at −80 °C.

### 2.4. PCR-HRM Analysis

The PCR was performed using the CFX Connect PCR detection system (Bio-Rad, Warsaw, Poland) and the KAPA HRM FAST qPCR Kit (Sigma Aldrich, St. Louis, MO, USA)—a ready-to-use mixture of components necessary for the PCR-HRM reaction with the EvaGreen dye. The composition of the reaction mixture was as follows: template DNA 4 μL; forward primer (10 μM)—0.6 μL; reverse primer (10 μM)—0.6 μL; deionized water added to 20 μL; KAPA HRM FAST qPCR Kit 10 μL. The primer sequences were as follows: R389G-F 5′TGGGCTACGCCAACTCGG3′, R 5′GGCCCCGACGACATCGTC3′, S49G-F 5′CCGGGCTTCTGGGGTGTTCC3′, R 5′GGCGAGGTGATGGCGAGGTAGC3′, CYP2D6*4F 5′ ACCCCTTACCCGCATCTC 3′ CYP2D6*4R 5′ TTGCTCACGGCTTTGTCC 3′. The thermal conditions of PCR were as follows: an initial denaturation at 95 °C for 3 min, followed by 40 cycles of 10 s at 95 °C, 10 s at 68 °C, and 10 s at 72 °C. For CYP2D6*4, initial denaturation at 95 °C for 10 s, followed by 40 cycles at 95 °C for 10 s, 10 s at 61 °C, and 10 s at 72 °C were used. After completion of the PCR, the obtained amplicon was heated in the temperature range of 70–90 °C with an increment rate of 0.1 °C/s. Bio-RadPrecision Melt Analysis™ v. 1.3 software was used to obtain normalized and differenced curves and calculate the value of the melting temperature of each PCR product.

Analysis of melt curves were performed using Precision Melt Analysis™ software v. 1.3 (Bio-Rad, Warsaw, Poland) by normalization and temperature-shifting of fluorescence data, followed by plotting of the difference in fluorescence. Data that are similar to each other were ‘clustered’ by the software and assigned a cluster number. The melt curves corresponding to each cluster were color-coded for easy visualization. The cluster detection settings include melt curve shape sensitivity (default value of 50% clustering) and melting temperature (T_m_) difference threshold (default of 0.15 degrees).

### 2.5. Statistical Analysis

Statistical analysis was conducted using Statistica PL v.13.0 software (Statsoft, Cracow, Poland). The χ^2^ test was performed with Yates’ correction. Statistical analyses were carried out between the study group and the control group, as well as between sex, age and types of arrhythmias (ventricular and supraventricular arrhythmias). Student’s *t*-test analyses were applied to assess differences in the data of patient characteristics. The data were presented as means and standard deviations (SD). The threshold for statistical significance was set at *p* < 0.05.

## 3. Results

### 3.1. Baseline Characteristics

The characteristics of the control group and the study group are presented in Table 1.

The group of 30 children with arrhythmia included 19 patients (63.3%) with ventricular arrhythmias (Va), among them 13 with ventricular extrasystoles (VES) and 6 with ventricular tachycardia (VT). Another 11 patients presented with supraventricular arrhythmias (SVa), either supraventricular tachycardia (SVT) (*n* = 8) or supraventricular extrasystoles (SVEs) (*n* = 3). Most patients from the arrhythmia group showed palpitations (*n* = 23); four reported chest pain, and five complained about frequent weakness. Other symptoms reported by patients from the arrhythmia group included dizziness and shortness of breath. Some patients from the arrhythmia group stated more than one symptom or ailment.

Two of the 20 persons in the control group were taking the following medications for reasons other than arrhythmia: ramipril at 2.5 mg once a day and amlodipine at 5 mg once a day. Twenty-one patients from the study group took the following drugs (dosage regimen has been established individually for each patient): metoprolol at 2 × 25 mg/2 × 12.5 mg/1 × 25 mg/1 × 47.51 mg/1 × 50 mg daily, sotalol at 2–20 mg per day, propafenone at 3 × 150 mg/150–75–75 mg/225–75–75 mg per day, propranolol at 3 × 40 mg daily, magnesium + vitamin B6 at 2 × 1 g/3 × 1 g/1 × 1 g (grams) per day, bisoprolol at 1–2.5 mg daily. One patient was taking sertraline at 1–50 mg daily and enalapril at 1–5 mg daily.

### 3.2. HRM Analysis

The selected polymorphisms were detected by PCR using the HRM technique.

#### 3.2.1. Detection of R389G Polymorphism

**Figure 3:**
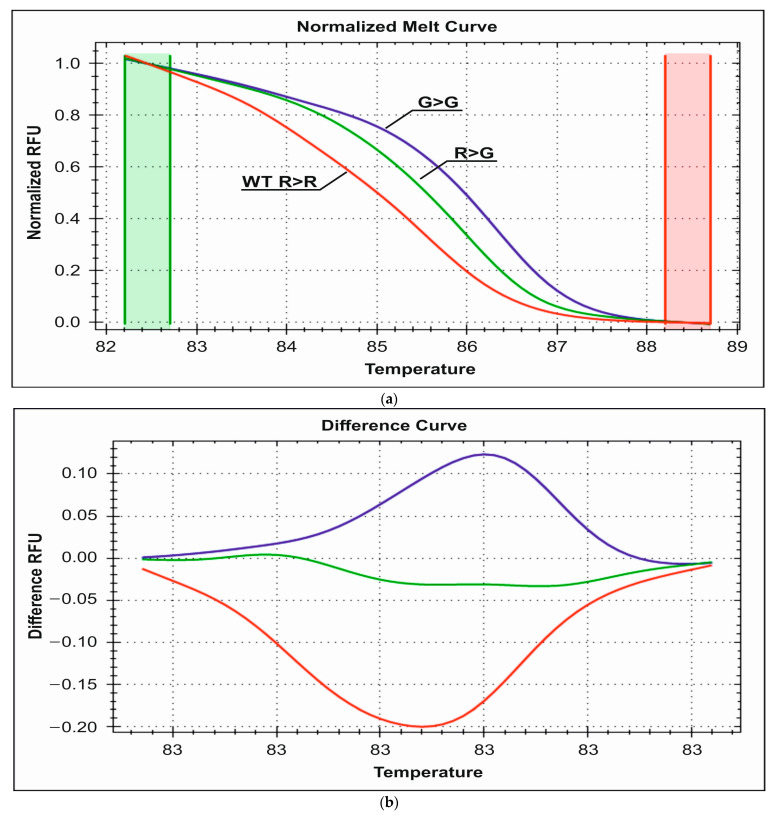
(**a**). Representative HRM normalized melting curves of the R389G polymorphism. (**b**) Difference plot melting curves of the R389 G genotyping. Red—wild R389R homozygous, green—heterozygous R389G, blue—homozygous mutant G389G.

HRM-PCR genotyping with the use of EvaGreen dye detected 12 R389G heterozygotes and three mutated G389G homozygotes in the control group and seven G389G homozygotes and 10 R389G heterozygotes in the study group. The rest of the individuals were wild-type homozygous for this locus. The obtained melting curves from the HRM analysis are presented in Figure 3a,b. The corresponding wild-type heterozygote and mutant homozygote melting temperatures are 85.5 °C, 86 °C, and 86.3 °C, respectively.

Using the Statistica software (v.13.0, Statsoft, Cracow, Poland). and the nonparametric χ^2^ test, it was shown that there was no statistically significant difference in the frequency of the R389G polymorphism between the control group and the study group (*p* > 0.05). In the study group divided into the type of cardiac arrhythmia (SVT or VT), it turned out that there was a statistically significant difference in the frequency of the R389G polymorphism between the control group and patients with SVT (*p* = 0.0002); the frequency of this polymorphism in the group of patients with SVT is statistically greater (0.54 vs. 0.45). A difference in the frequency of this polymorphism was demonstrated between the control group and patients with VT arrhythmias (*p* = 0.0002); these patients have a lower frequency of this polymorphic change compared to the control group (0.31 vs. 0.45). There was also a statistically significant difference in the frequency of the R389G polymorphism between both groups of patients with SVT and VT lesions (*p* = 0.0004, 0.54 vs. 0.31). Statistically significant differences in the frequency of the studied polymorphic changes were also shown between the sexes: girls from the group of patients with SVT and VT differed significantly in terms of the frequency of polymorphic changes (*p* = 0.007, 0.57 vs. 0.33), with the frequency of this change being significantly greater in the group of girls with arrhythmia types of SVT. Such a relationship was not observed for the boys from the test group (*p* > 0.05); see Table 2.

#### 3.2.2. Detection of S49G Polymorphism

**Figure 4:**
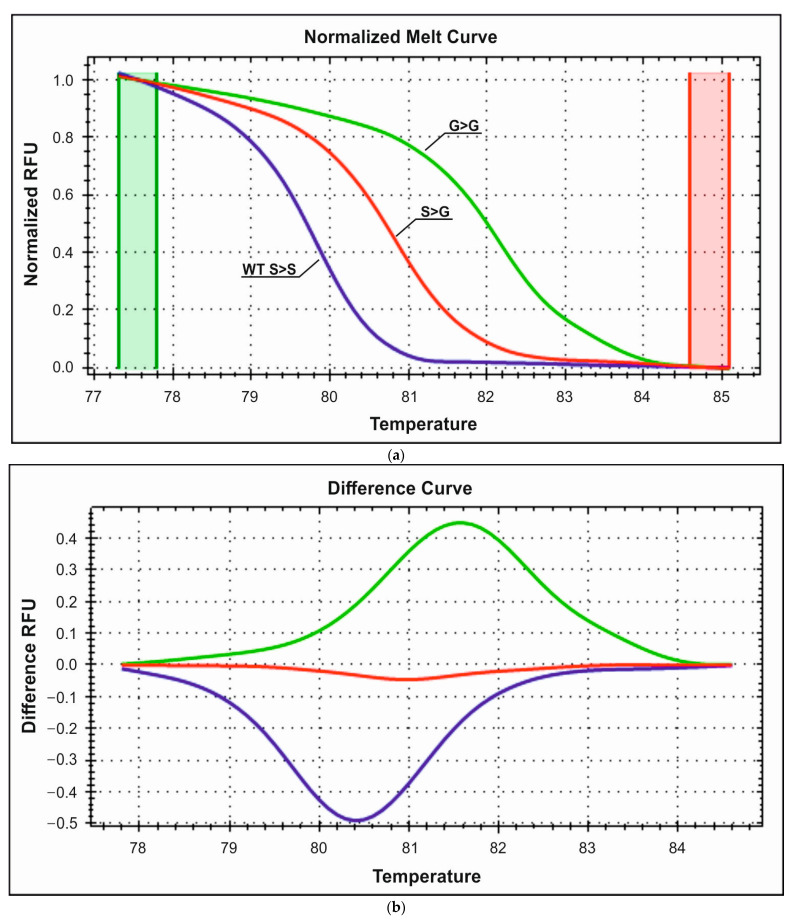
(**a**) Representative HRM normalized melting curves of the S49G polymorphism. (**b**) Difference plot melting curves of the S49G genotyping. Blue—wild S49S homozygous; red—heterozygous S49G; green—homozygous mutant G49G.

The analysis of the S49G polymorphism revealed the presence of one G49G mutant homozygote and three S49G heterozygotes in the control group and five S49G heterozygotes and one G49G homozygote in the study group. The obtained melting curves are presented in Figure 4a,b. The corresponding wild-type heterozygote and mutant homozygote melting temperatures are 80.9 °C, 81.1 °C, and 81.8 °C, respectively.

Statistical analysis of the S49G polymorphism showed no significant difference in the frequency of its occurrence between the control group and the study group (*p* > 0.05), and patients with SVT and VT arrhythmias (*p* > 0.05, 0.09 vs. 0.13). Moreover, a statistically significant difference in the frequency of this polymorphism was not found for both girls and boys with SVT and VT arrhythmias (*p* > 0.05); see Table 3.

#### 3.2.3. Detection of CYP2D6*4 Polymorphism

**Figure 5:**
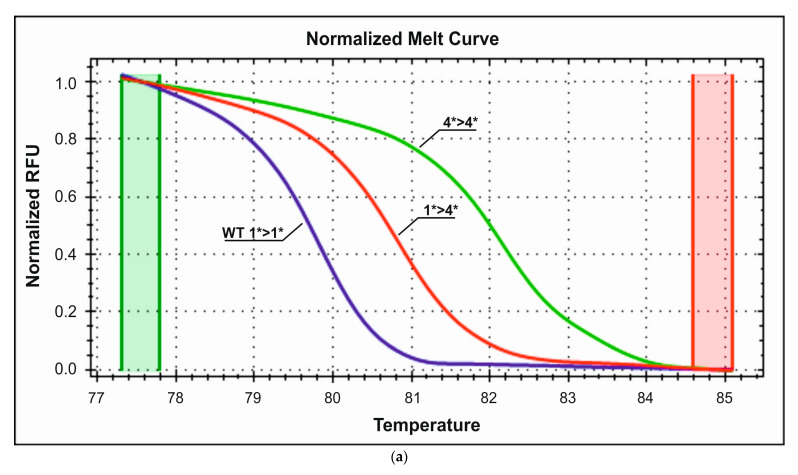

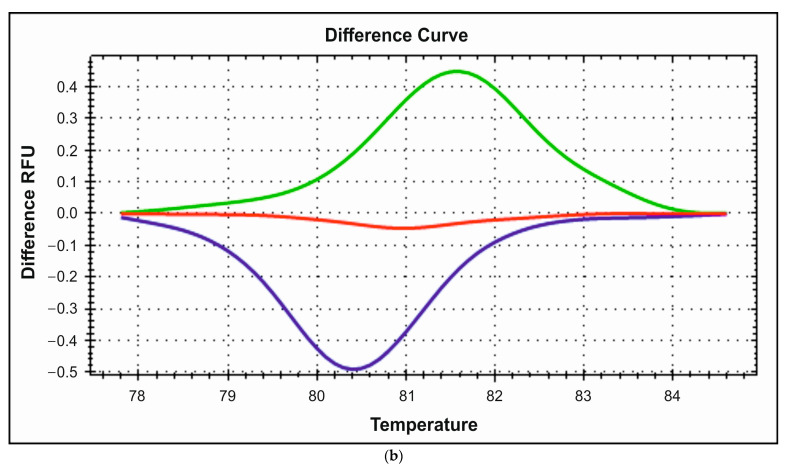
(**a**). Representative HRM normalized melting curves of the *CYP2D6* polymorphism. (**b**) Difference plot melting curves of the *CYP2D6* genotyping. Blue–wild *1/*1 homozygous; red—heterozygous *1/*4; green—homozygous mutant *4/*4.

In the control group, the genetic analysis detected three carriers of the heterozygous *CYP2D6* allele 4 and two individuals homozygous for this locus. The study group demonstrated a higher frequency of the *CYP2D6* allele 4 (0.28). Genetic analysis revealed the presence of four people homozygous for this locus and nine who are heterozygous system carriers. The obtained melting curves are presented in Figure 5a,b. The corresponding wild-type heterozygote and mutant homozygote melting temperatures are 85.4 °C, 86 °C, and 86.2 °C, respectively.

Statistical analysis using the χ^2^ test showed no statistically significant differences between the study group and the control group in the frequency of the CYP2D6*4 allele (*p* > 0.05); see Table 4.

## 4. Discussion

The occurrence of genetic polymorphisms affects the effectiveness of the therapies used. The possibility of genotyping enables the assessment of the patient’s response to the drug. This offers an opportunity to personalize the therapy so that it brings the best results while minimizing side effects.

The study aimed to detect selected polymorphisms in the β1-adrenergic receptor and CYP2D6*4 polymorphism using the PCR-HRM technique, as well as to assess the frequency of their occurrence in the control and study groups. The high-specificity HRM technique is commonly used to detect of polymorphisms known in the literature [21,42,43].

The material used during the study was the blood of 50 patients from the Department and Clinic of Pediatric Cardiology. Among them the study group consisted of 30 participants who were treated for ventricular (VT) or/and supraventricular (SVT) arrhythmias and the control group (free of arrhythmias) consisted of 20 individuals. Nucleotide changes in the studied polymorphic sites change the melting point of the obtained amplicons. Based on the profiles of the melting point curves (after their normalization using the BioRad software v.1.3), it is possible to determine the genotype of each sample. The HRM analysis concerned the two most common polymorphisms in the β-1 adrenergic receptor, R389G and S49G, which allowed the detection of carriers of heterozygous and homozygous system in the study group and the control group.

Arg389 homozygotes show greater activity and more effective signaling compared to Gly389 [44]. In addition, Arg389 homozygotes were associated with a better response to β-blockers in the treatment of hypertension, glaucoma (large reduction in intraocular pressure), and heart failure (large improvement in LVEF, better drug tolerance) compared to Gly389. Arg389Arg showed a good response to treatment with metoprolol [45]. Arg389 homozygotes are characterized by a higher left ventricular mass index (LVMI) and greater diastolic septal thickness (IVSd). The Gly389 allele is more common in black populations [46].

It has been found that the presence of the Gly389 allele and the Gly389 homozygote increases the risk of heart failure (HF) in East Asian populations and reduces the risk of HF in white populations [47].

The effect of Ser49Gly polymorphism on the cardiovascular system has been demonstrated. In a study by Mahesh Kumar et al. [48], 41 healthy male volunteers subjected to preliminary genetic testing were divided according to the polymorphisms present, i.e., S49S, R389R, S49G, R389R, G49G R389R, S49S R389G, S49S G389G, and S49G R389G. Each group performed exercises on the treadmill. Cardiovascular parameters were measured before and after metoprolol administration. It was shown that, in the S49S/G389G group, the diastolic blood pressure was lower than in the S49S/A389A group. There were no differences in systolic and diastolic blood pressure between Ser49Gly polymorphisms. The tests performed before and after drug administration did not differ significantly [48]. The impact of polymorphism on the performance of athletes was also assessed by Marek Sawczuk et al. [49]. It covered 223 Polish athletes and 357 volunteers. The Gly49 variant or the Gly49: Arg389 haplotype has been observed to have a positive effect on sports performance due to a beneficial effect on heart function [49].

The Gly49 allele reduces the density of the β1-adrenergic receptor, which results in a lower accumulation of cAMP and a slower heart rate, hence the cardioprotective effect of the polymorphism. The presence of Gly49Gly homozygotes in patients with HF is associated with better clinical outcomes and a lower risk of mortality [50]. On the other hand, homozygous Ser49 influences increased adrenergic activity [51]. A slight decrease in heart rate was observed in response to carvedilol in Arg389 homozygotes [52].

Rong Gu et al. [53] indicated that Gly389 carriers are more sensitive to the effects of bisoprolol and have a better prognosis compared to Arg389 homozygotes. Patients with the Gly389 genotype tend to achieve better therapeutic results. On the other hand, patients with the Arg389 genotype require a higher dose of β-blockers. The Ser49Ser genotype responded less to β-blockers and required higher drug doses. The study showed that the Ser49Gly polymorphism has no apparent effect on the pharmacotherapy used, while the Arg389Gly polymorphism is more related to the therapeutic effect of drugs [53].

There is a relationship between the occurrence of arrhythmias and gender. Supraventricular arrhythmias are more common in women. It is associated with the female sex hormones estrogen and progesterone, which increase the sensitivity of cardiomyocytes to endogenous agonists [54]. Estrogen reduces the density of L-type calcium channels. It also affects the expression of β-adrenergic receptors. In premenopausal women, it declines with age but stabilizes after menopause. No relationship was found between age and ADRB1 expression in men. Moreover, estrogen receptors are located in the heart, and their molecular similarity to ADRB1 means that beta-blockers may interact with them [55]. The phase of the menstrual cycle also influences the incidence of supraventricular arrhythmias. In the luteal phase, when the progesterone concentration is the highest and estrogen is the lowest, there is a high probability of tachycardia [54]. Ventricular arrhythmias are more often diagnosed in men than women. This is related to the higher incidence of heart failure in men, which may also be caused by another medical condition, for example, coronary heart disease (CAD), heart inflammation, hypertension, cardiomyopathy [56]. Despite the presented differences, the pharmacotherapy of women does not differ from generally defined treatment standards [57].

CYP2D6 accounts for a small percentage of all hepatic CYPs but is responsible for the metabolism of approximately 25% of all drugs metabolized by CYPs [58]. The *CYP2D6* gene is highly polymorphic, with more than 60 variant alleles (http://www.cypalleles.ki.se accesed on 3 January 2022). Several of these variants encode an inactive protein (e.g., *3, *4, *5, *6), and patients with two nonfunctional alleles are classified as “poor metabolizers” (PMs), while carriers of one or two functional alleles (*1, *2) are classified as “extensive metabolizers” (EMs). Approximately 5–10% of Caucasian populations are PMs [27,31,35,36,59,60]. Subjects with one nonfunctional and one functional allele can also be considered “intermediate metabolizers” (IMs). CYP2D6*4 (rs3892097 G > A) is the most common variant allele (allele frequency of 20%) in Caucasians and is the most frequent nonfunctional allele in the PM phenotype; over 75% of the PMs are carriers of this polymorphism [59]. The genetic status of CYP2D6 may be a determinant of beta-blocker tolerability [61,62].

However, the percentage of individuals carrying the *CYP2D6 4* allele as either homozygous or heterozygous was significant (17 alleles out of 30 in the test group and 7 alleles out of 20 in the control group. The presence of the 4 allele of *CYP2D6* impacts slower metabolism and prolongs the half-life of beta-1 blockers used in the treatment of patients. Genetic testing of patients was performed after the pharmacological treatment of patients, therefore, they did not affect the selection of the dose of several beta-1 adrenergic receptor antagonists used in the treatment of cardiac arrhythmias. Nevertheless, the observed high frequency of the studied polymorphism should be taken into account when selecting the dose of the beta-1 blocker in optimizing the pharmacotherapy of the new group of patients.

The Arg389Gly mutation in the beta 1 adrenergic receptor may affect the effectiveness and response to beta-blockers. This mutation is associated with differences in receptor interaction with beta-adrenergic drugs. Individuals with this mutation may have a decreased sensitivity to certain beta-blockers compared to those without the mutation. Homogeneity for Arg389Arg in the case of mutations in the beta-1 adrenergic receptor gene may be associated with increased sensitivity to certain beta-blockers. This means that individuals who are homozygotes of Arg389Arg may need lower doses of beta-blockers compared to those without this mutation to the desired therapeutic effect. Individuals carrying *CYP2D6* allele 4 may have a reduced ability to transform beta-blockers, which may result in prolonged life of these drugs and an increased risk of side effects. Therefore, it is important to adjust the dosage of beta-blockers to the individual needs and tolerance of the patient, as well as to monitor side effects. However, the impact of the Arg389Gly mutation on the dosage of beta-blockers is complex and may depend on many factors, such as the type of drug, the severity of the symptoms of the disease, the general health of the patient, etc. The dosage of beta-blockers should always be adjusted individually by a doctor who takes into account all these factors, including the presence of such genetic mutations [1,24,63,64].

The HRM technique is highly sensitive and enables quick detection of genetic polymorphisms. An additional advantage is the low cost of the analysis. The method of combining PCR and HRM used in the work protects against the contamination of samples. Its relative simplicity also prevents many analytical errors. Therefore, the above technique could be employed to personalize pharmacotherapy. Currently, such studies are not commonly performed. However, the examples of polymorphisms presented in this paper show that knowing the patient’s genetic profile could improve the therapeutic process. To sum up, the method used in this work is a quick and relatively cheap way to detect polymorphisms and develop pharmacotherapy.

## 5. Conclusions

In conclusion, the genotyping of the two polymorphic sites Arg389Gly and Ser49Gly in the β1-adrenergic receptor gene in pediatric patients diagnosed with cardiac arrhythmias led to the detection of mutant heterozygous and homozygous system carriers in both the study and control groups. The genetic analysis showed a statistically significant difference in the incidence of Arg389Gly polymorphism between patients with SVT and VT arrhythmias in the study group and patients in the control group. In addition, statistical differences in the frequency of the analyzed Arg389Gly polymorphism alleles between patients with SVT and VT arrhythmias were also found. Furthermore, compared to females with VT, the group of girls with SVT arrhythmia has a statistically higher prevalence of Arg389Gly polymorphism. A few carriers of homozygous and heterozygous systems of the S49G polymorphism were observed in both the control group and the group of patients experiencing arrhythmias. In both the test and control groups, there was a substantial difference in the proportion of people with the CYP2D6*4 allele who were homozygous or heterozygous. Because CYP2D6*4 allele carriers are highly prevalent in both groups, beta-1 blocker therapy needs to be optimized.

### Limitations of the Study

Our study has several limitations. First, the sample was not large and all patients were recruited at the same center. To finally draw conclusions regarding the diagnostic accuracy of the analyzed polymorphisms in patients with arrhythmia, a multicenter, randomized, prospective study on a large sample is necessary. Second, although our control group consisted of “nonarrhythmic” subjects, they were not free from other (non-cardiological) symptoms and comorbidities. For this reason, it seems that in the preparation of the next manuscript, it is worth creating a control group of completely healthy volunteers to comprehensively analyze the diagnostic accuracy of the analyzed polymorphisms in the screening and differential diagnosis of Va and SVa. In summary, further studies using a unified model are needed to definitively draw conclusions about the potential role of the discovered polymorphisms in the pathogenesis of idiopathic arrhythmias in children. Nevertheless, even in a study group of 30 patients, we observed some differences in results compared to the control group. This is a serious incentive for us to include more topics in the next manuscript.

## Figures and Tables

**Table 1:** General characteristics of patients with arrhythmias and control group.

General Characteristics	Study Group (*N* = 30)	Control Group (*N* = 20)	*p*-Value
Gender (F/M)	16/14	12/8	-
Mean age (years)	15.5 ± 1.9	15.8 ± 1.2	0.59
Height (cm)	169.7 ± 9.7	167.4 ± 5.7	0.34
Weight (kg)	63.8 ± 18.3	63.5 ± 11.7	0.94
BSA (m^2^)	1.72 ± 0.24	1.71 ± 0.15	0.81
BMI	21.9 ± 5.2	22.4 ± 3.6	0.71
BP syst (mmHg)	117.5 ± 10.4	120.5 ± 14.5	0.40
BP diast (mmHg)	64.9 ± 8.0	62.6 ± 0.6	0.39
Sat O_2_ (%)	98.3 ± 0.7	98.4 ± 1.1	0.79
Laboratory tests			
CK-MB (U/L)	14.1 ± 5.3	12.1 ± 5.8	0.35
WBC (10^3^ u/L)	6.49 ± 1.83	6.88 ± 2.08	0.50
RBC (10^6^ u/L)	4.88 ± 0.45	4.80 ± 0.47	0.53
HGB (g/dL)	13.08 ± 1.31	14.0 ± 1.25	0.61
HCT (%)	41.5 ± 2.7	41.1 ± 3.8	0.62
TSH (mL U/L)	2.43 ± 1.05	2.36 ± 1.27	0.85
FT4 (ng/dL)	1.3 ± 0.18	1.4 ± 0.39	0.14
Echocardiography parameters			
LVIDD (mm)	48.7 ± 4.5	48.5 ± 3.1	0.86
LVIDS (mm)	31.7 ± 13.7	28.8 ± 3.3	0.38
LV-EF (%)	68.7 ± 7.9	69.9 ± 5.9	0.59
LV-SF (%)	40.7 ± 6.1	39.9 ± 4.9	0.63
Exercise test			
Stress test (gr)	3.7 ± 0.6	3.8 ± 0.5	0.67
METS	11.2 ± 1.1	11.4 ± 0.73	0.56
NYHA scale (I/II)	22/8	18/2	-

Abbreviations: BSA—body surface area; BMI—body mass index; BP syst/diast—systolic (syst) and diastolic (diast) blood pressure (BP); Sat O_2_ (%)—oxygen saturation; CK-MB—creatine kinase-MB fraction; WBC—white blood cells—leukocytes; RBC—red blood cells—erythrocytes; HGB—hemoglobin; HCT—hematocrit; TSH—thyroid hormone; FT4—thyroxine; LVIDD—left ventricular internal diameter end diastole; LVIDS—left ventricular internal diameter at end systole; LV-EF—left ventricle ejection fraction; LV-SF—left ventricular systolic function; METS—metabolic equivalents (METS) in exercise testing; NYHA Classification—Stages of Heart Failure: Class I—no symptoms and no limitation in ordinary physical activity; e.g.,; shortness of breath when walking; climbing stairs; etc. Class II—mild symptoms (mild shortness of breath and/or angina) and slight limitation during ordinary activity.

**Table 2:** R389G polymorphism of patients with arrhythmias and control group.

R389G Polymorphism											
Study Group						Control Group					
	Genotype			Allele Frequency		Genotype				Allele Frequency	
Type of Arrhythmia	RR	RG	GG	R	G		RR	RG	GG	R	G
VT+SVT (*n* = 30)	13	10	7	36 (0.6)	24 (0.4)						
VT (*n* = 19)	9	8	2	26 (0.69)	12 (0.31)	(*n* = 20)	5	12	3	22 (0.55)	18 (0.45)
SVT (*n* = 11)	4	2	5	10 (0.46)	12 (0.54)						
χ^2^	14.2	*p* = 0.0002	SVT vs. control								
	6.45	*p* = 0.0002	VT vs. control								
	12.5	*p* = 0.0004	SVT vs. VT								
gender											
female (*n* = 16)	7	4	5	18 (0.56)	14 (0.44)	(*n* = 12)	3	8	1	14 (0.58)	10 (0.42)
SVT female (*n* = 7)	3	0	4	6 (0.43)	8 (0.57)						
VT female (*n* = 9)	4	4	1	12 (0.67)	6 (0.33)						
male (*n* = 14)	6	6	2	18 (0.64)	10 (0.36)	(*n* = 8)	2	4	2	8 (0.5)	8 (0.5)
χ^2^	3.12	*p* = 0.007	SVT girls vs. VT girls								
age											
11–14 (*n* = 10)	4	5	1	13 (0.65)	7 (0.35)	11–14 (*n* = 3)	0	2	1	2 (0.33)	4 (0.67)
15–16 (*n* = 6)	2	1	3	5 (0.42)	7 (0.58)	15–16 (*n* = 9)	2	6	1	10 (0.56)	8 (0.44)
16.5–18 (*n* = 14)	7	4	3	18 (0.64)	10 (0.36)	16.5–18 (*n* = 8)	3	4	1	10 (0.63)	6 (0.37)

**Table 3:** S49G polymorphism of patients with arrhythmias and control group.

S49G Polymorphism											
Study Group						Control Group					
	Genotype			Allele Frequency		Genotype				Allele Frequency	
Type of Arrhythmia	SS	SG	GG	S	G		SS	SG	GG	S	G
VT + SVT (*n* = 30)	24	5	1	53 (0.88)	7 (0.12)						
VT (*n* = 19)	15	3	1	33 (0.87)	5 (0.13)	(*n* = 20)	16	3	1	35 (0.88)	5 (0.12)
SVT (*n* = 11)	9	2	0	20 (0.91)	2 (0.09)						
gender											
female (*n* = 16)	12	3	1	27 (0.84)	5 (0.16)	(*n* = 12)	10	1	1	21 (0.875)	3 (0.125)
male (*n* = 14)	12	2	0	26 (0.93)	2 (0.07)	(*n* = 8)	6	2	0	14 (0.875)	2 (0.125)
age											
11–14 (*n* = 10)	9	1	0	19 (0.95)	1 (0.05)	11–14 (*n* = 3)	3	0	0	6 (1.00)	0 (0)
15–16 (*n* = 6)	4	2	0	10 (0.83)	2(0.17)	15–16 (*n* = 9)	7	2	0	16 (0.89)	2 (0.11)
16.5–18 (*n* = 14)	11	2	1	24 (0.86)	4 (0.14)	16.5–18 (*n* = 8)	6	1	1	13 (0.81)	3 (0.19)

**Table 4:** CYP2D6*4 polymorphism of patients with arrhythmias and control group.

CYP2D6*4 Polymorphism									
Study Group					Control Group				
Genotype				Allele Frequency	Genotype				Allele Frequency
Type of Arrhythmia	WT/WT	WT/*4	4/4	4*		WT/WT	WT/*4	4/4	4*
VT+SVT (*n* = 30)	17	9	4	17 (0.28)					
VT (*n* = 19)	11	4	4	12 (0.31)	(*n* = 20)	15	3	2	7 (0.17)
SVT (*n* = 11)	6	5	0	5 (0.23)					
gender									
female (*n* = 16)	10	4	2	8 (0.25)	(*n* = 12)	10	1	1	3 (0.125)
male (*n* = 14)	7	5	2	9 (0.32)	(*n* = 8)	5	2	1	4 (0.25)
age									
11–14 (*n* = 10)	7	3	0	3 (0.15)	11–14 (*n* = 3)	3	0	0	0
15–16 (*n* = 6)	2	2	2	6 (0.5)	15–16 (*n* = 9)	5	3	1	5 (0.28)
16.5–18 (*n* = 14)	8	4	2	8 (0.28)	16.5–18 (*n* = 8)	7	0	1	2 (0.125)

## Data Availability

The data are unavailable due to privacy and ethical restrictions. The data presented in this study are available upon request from the corresponding author.
